# Epstein-Barr Virus–Positive Lymphoepithelioma-Like Carcinoma in Celiac Disease

**DOI:** 10.14309/crj.0000000000000970

**Published:** 2023-02-09

**Authors:** Robert Kwei-Nsoro, Yuchen Wang, Seema Gandhi, Pius Ojemolon, Moyosoluwa Awoyomi, Annabel Ogar

**Affiliations:** 1 Department of Medicine, John H. Stroger, Jr Hospital of Cook County, Chicago, IL; 2 Division of Gastroenterology, John H. Stroger, Jr Hospital of Cook County, Chicago, IL

**Keywords:** lymphoepithelioma-like carcinoma, celiac disease, Epstein-Barr virus

## Abstract

Lymphoepithelioma-like carcinoma (LELC) is a rare lymphoproliferative malignancy that has been described in many organs over the years. LELC in the duodenum has rarely been described in literature. This article aims to present a rare cause of melena in a young man and the diagnostic challenge that ensued to throw more light on this rare disease. In this article, we describe a 43-year-old man who presented with melena and weight loss and was subsequently diagnosed with LELC after multiple endoscopic biopsies. The patient was also found to have celiac disease in association with his LELC.

## INTRODUCTION

Lymphoepithelioma is a form of undifferentiated carcinoma initially described in nasopharyngeal carcinomas.^1^ Cancers with similar histological features that occur outside the nasopharynx are called lymphoepithelioma-like carcinoma (LELC). LELC has been described in organs such as the stomach, lung, bladder, esophagus, thymus, cervix, skin, and salivary glands.^2–10^ There have only been 2 documented cases in literature of LELC in the duodenum, and both were described in association with Epstein-Barr virus (EBV).^11,12^

## CASE REPORT

A 43-year-old Eastern European man with no significant medical history presented with melena and unintentional 20-pound weight loss. Work-up revealed severe anemia which was managed with blood transfusions and an urgent esophagogastroduodenoscopy.

**Figure 1.**
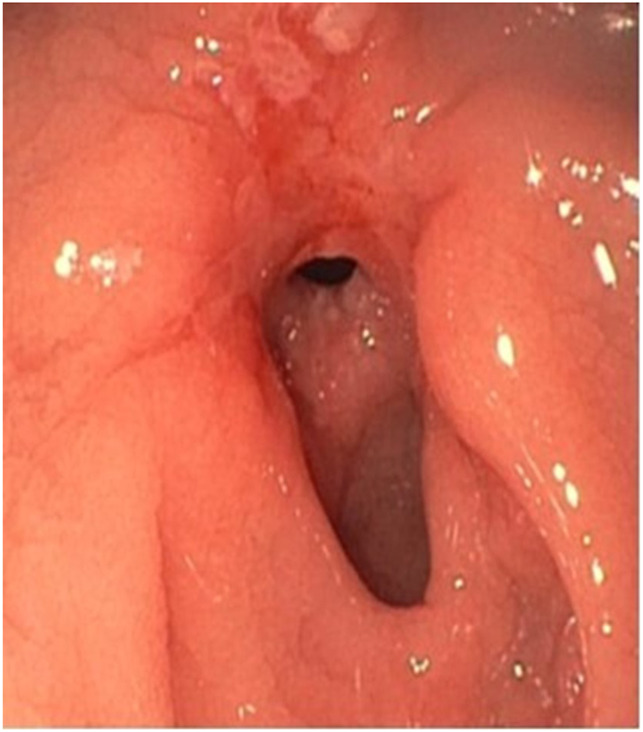
Esophagogastroduodenoscopy showing circumferential narrowing at the level of the duodenal sweep.

**Figure 2.**
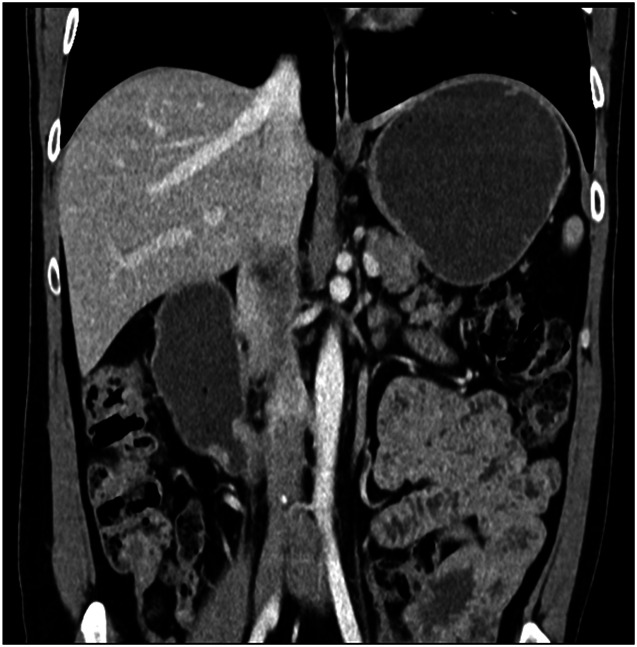
Computed tomography scan showing dilation of the stomach and duodenal bulb with an abrupt narrowing at the duodenal sweep.

**Figure 3.**
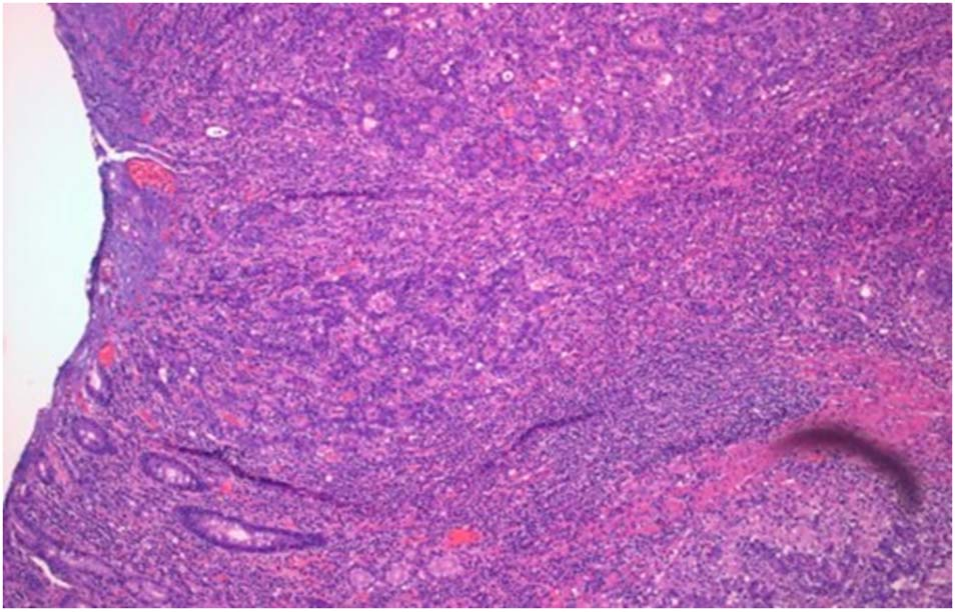
(300 dpi) At low power, shows histology of the stricture with abundant lymphoid infiltrate.

**Figure 4.**
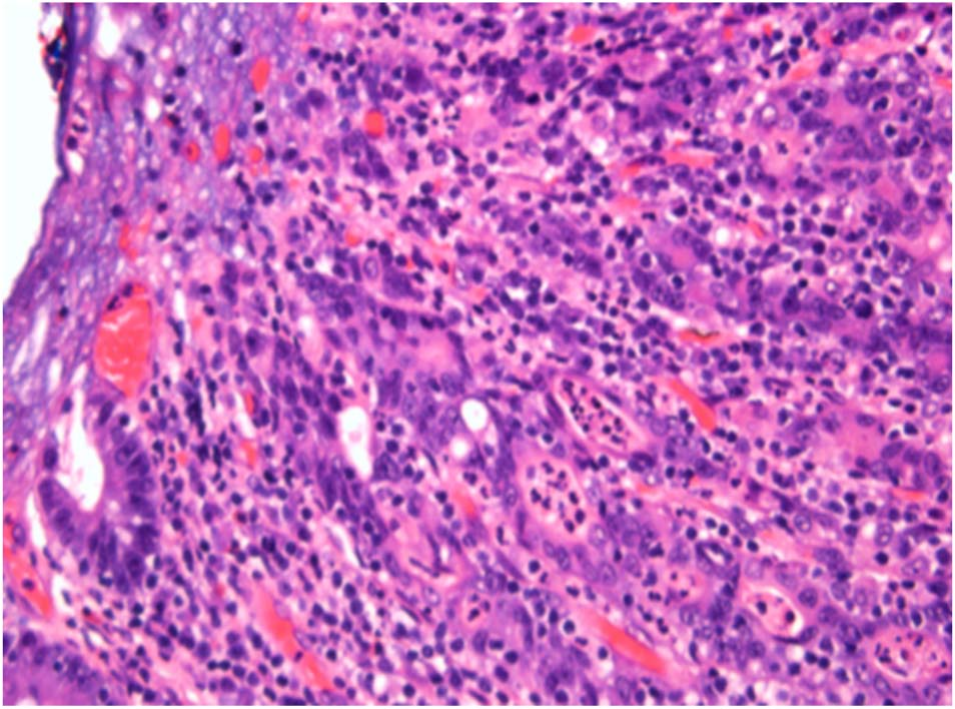
(300 dpi) At high power, shows multiple squamoid nests with hyperchromatic chromatin and prominent nucleoli and a lymphoplasmacytic infiltrate.

Esophagogastroduodenoscopy revealed a dilated stomach with retained solid food and a dilated duodenal bulb with circumferential narrowing at the level of the duodenal sweep (Figure 1). Proximal to the circumferential stricture, there was a 1-cm ulceration with a bleeding visible vessel. Hemostasis was achieved with epinephrine and hemoclips. The endoscope was able to easily pass through the stricture, and the ampulla was seen to be normal distal to the stricture. The surrounding mucosa was extremely friable and granular in appearance. Initial endoscopic biopsy results showed chronic inflammation and prominent atypical glandular proliferation. Over the next month, multiple endoscopies were performed to re-evaluate the stricture and obtain biopsies because of its abnormal appearance and inconclusive biopsy results. A computed tomography scan of the abdomen performed during work-up revealed dilation of the stomach and duodenal bulb and an abrupt narrowing at the duodenal sweep (Figure 2). After repeated endoscopies, the histology of the stricture revealed multiple squamoid nests with hyperchromatic chromatin and prominent nucleoli, separated from each other by an abundant lymphoid infiltrate. Staining was positive for keratin AE1/AE3, CDX-2, and CK20, and immunostaining was positive for EBV (Figures 3 and 4). Beta-catenin showed membranous staining and there was intact nuclear expression for MLH1, MSH2, MSH6, and PMS2. In addition, stains for TTF-1, CD45, CK7, CD31, CD34, p40, and NKX3.1 were negative. The patient remained well during this period and was scheduled for pancreaticoduodenectomy, which showed an ulcerated pink-tan firm mass (2.2 × 1.4 × 0.4 cm in maximum depth) in the midportion of the duodenum with a corresponding stricture (4.5 cm in circumference × 0.5 cm in length) directly distal to the mass and located 7.0 cm from the nearest distal margin. On sectioning, the mass had a tan-white fibrous cut surface extending through the muscularis layer of the duodenum without further involvement of the surrounding structures. The duodenal mucosa proximal to the mass had a diffuse nodular appearance and was slightly dilated, and the duodenal mucosa distal to the mass had marked thinning of its wall. There were no other grossly suspicious masses or lesions. Pathology showed invasive adenocarcinoma of the duodenum with lymphoepithelioma-like features involving the muscularis propria with no lymphovascular or perineural invasion and negative resection margins as well. There was no evidence of distance metastasis, and the tumor was thus staged T2N0M0. The patient got no adjuvant chemotherapy and remains cancer-free 18 months after the surgery.

Histology of the biopsies from the distal duodenum revealed chronic active duodenitis with villous blunting, increased intraepithelial lymphocytes, and mild active inflammation. Tissue transglutaminase (endomysial) immunoglobulin A was found to be elevated to 64.0 U/mL consistent with celiac disease (CD). The patient was started on a gluten-free diet and has remained asymptomatic during scheduled follow-ups.

## DISCUSSION

LELC is a rare type of malignancy that is characterized by proliferation of poorly differentiated malignant cells within a prominent lymphoid infiltrate.^10^ To the best of our knowledge, this is the first case of LELC in the duodenum being described in the United States. Two other cases have, however, been described in Italy and Spain.^11,12^ The first ever case of LELC in the duodenum was described in a 63-year-old patient with active Crohn's disease after presenting with severe anemia and intestinal subobstructive symptoms.^11^ The second case was described in a 77-year-old man who was undergoing work-up for chronic microcytic anemia in Spain.^12^ Immunohistochemical analysis was also positive for EBV in both patients. Many observational studies have suggested a causal relationship between LELC and EBV, but this is mostly true for upper aerodigestive tract LELC.^2–10^ Abnormalities involving p53 regulation and human papillomavirus have been described in association with LELC in the urinary tract and cervix, respectively.^7,13,14^ Immunohistochemical analysis, polymerase chain reaction, and in situ hybridization have confirmed the presence of EBV DNA and RNA in malignant epithelial cells of LELC.^15,16^ Of particular interest is the Epstein-Barr–encoded RNA, which has been shown to be abundantly expressed in malignant epithelial cells of LELC.^10^ In addition to the 2 other cases described of LELC in the duodenum, our report further supports the oncogenic role of EBV in LELC arising in the duodenum.

Interestingly, the cancer reported here occurred in a patient with CD. CD has never been described in association with LELC but has been shown to increase the risk of developing adenocarcinoma and lymphoproliferative malignancies.^17^ The association between CD and small bowel adenocarcinoma (SBA) was first described more than 40 years ago.^18^ The link between SBA and CD has been explored in a few case reports, implying that a variety of mechanisms may contribute to the pathogenesis of SBA arising in the setting of CD.^19,20^ These mechanisms include chronic inflammation, increased permeability of carcinogenic factors, malabsorption of anticarcinogenic substances, as well as impaired immune surveillance.^19,20^ Having established the oncogenic properties of both EBV and CD, it is entirely plausible to suggest that the combined presence of EBV and CD in this patient likely played a huge role in his cancer diagnosis.

Treatment of LELC is mainly surgical with or without adjuvant chemotherapy. Our patient was T2N0M0 at diagnosis and required no adjuvant chemotherapy after pancreaticoduodenectomy. He remains cancer-free at 18 months similar to the 2 other patients. Because of our very limited sample size, it is inherently difficult to make any conclusion regarding prognosis; however, gastric LELC which is more common compared with duodenal LELC has been shown to have a better overall prognosis compared with the other gastric tumor subtypes.^21–23^ The improved survival has been suggested to be due to reduced tumor spread from the intense lymphocytic proliferation associated with LELC.^10^

## DISCLOSURES

Author contributions: All authors participated in the writing and review of the manuscript. R. Kwei-Nsoro is the article guarantor.

Financial disclosure: None to report.

Informed consent was obtained for this case report.

## References

[1] Chan JYK Wong EWY Ng SK Vlantis AC. Non-nasopharyngeal head and neck lymphoepithelioma-like carcinoma in the United States: A population-based study. Head Neck. 2016;38(Suppl 1):E1294–300. 26316257 10.1002/hed.24215

[2] Tamas EF Nielsen ME Schoenberg MP Epstein JI. Lymphoepithelioma-like carcinoma of the urinary tract: A clinicopathological study of 30 pure and mixed cases. Mod Pathol. 2007;20(8):828–34. 17541442 10.1038/modpathol.3800823

[3] Pavithra Herath CH Chetty R. Epstein-Barr virus-associated lymphoepithelioma-like gastric carcinoma. Arch Pathol Lab Med. 2008;132(4):706–9. 18384225 10.5858/2008-132-706-EVLGC

[4] Bildirici K Ak G Peker B . Primary lymphoepithelioma-like carcinoma of the lung. Tuberk Toraks. 2005;53(1):69–73. 15765291

[5] Chen PCH Pan CC Hsu WH Ka HJ Yang AH. Epstein-Barr virus-associated lymphoepithelioma-like carcinoma of the esophagus. Hum Pathol. 2003;34(4):407–10. 12733124 10.1053/hupa.2003.71

[6] Sun XN Xu J Yang QC Hu JB Wang Q. Lymphoepithelioma-like carcinoma of the submandibular salivary gland: A case report. Chin Med J. 2006;119(15):1315–7. 16919194

[7] Bais AG Kooi S Teune TM Ewing PC Ansink AC. Lymphoepithelioma-like carcinoma of the uterine cervix: Absence of Epstein-Barr virus, but presence of a multiple human papillomavirus infection. Gynecol Oncol. 2005;97(2):716–8. 15863191 10.1016/j.ygyno.2005.01.016

[8] Cavalieri S Feliciani C Massi G . Lymphoepithelioma-like carcinoma of the skin. Int J Immunopathol Pharmacol. 2007;20(4):851–4. 18179760 10.1177/039463200702000424

[9] Leyvraz S Henle W Chahinian AP . Association of Epstein-Barr virus with thymic carcinoma. N Engl J Med. 1985;312(20):1296–9. 2985993 10.1056/NEJM198505163122006

[10] Kijima Y Hokita S Takao S . Epstein-Barr virus involvement is mainly restricted to lymphoepithelial type of gastric carcinoma among various epithelial neoplasms. J Med Virol. 2001;64(4):513–8. 11468737 10.1002/jmv.1079

[11] Vanoli A Di Sabatino A Biancone L . Small bowel Epstein-Barr virus-positive lympho-epithelioma-like carcinoma in Crohn's disease. Histopathology. 2017;70(5):837–9. 27891660 10.1111/his.13133

[12] Chaparro Mirete M López-López V Robles Campos R. Lymphoepithelioma-like carcinoma of the duodenum: A very infrequent tumor. Rev Esp Enferm Dig. 2020;112(3):239. 32022569 10.17235/reed.2020.6503/2019

[13] Gulley ML Burton MP Allred DC . Epstein-Barr virus infection is associated with p53 accumulation in nasopharyngeal carcinoma. Hum Pathol. 1998;29(3):252–9. 9496828 10.1016/s0046-8177(98)90044-2

[14] Chen W Cooper NR. Epstein-Barr virus nuclear antigen 2 and latent membrane protein independently transactivate p53 through induction of NF-kappaB activity. J Virol. 1996;70(7):4849–53. 8676521 10.1128/jvi.70.7.4849-4853.1996 PMC190431

[15] Wong MP Chung LP Yuen ST . In situ detection of Epstein-Barr virus in non-small cell lung carcinomas. J Pathol. 1995;177(3):233–40. 8551384 10.1002/path.1711770304

[16] Higashiyama M Doi O Kodama K . Lymphoepithelioma-like carcinoma of the lung: Analysis of two cases for Epstein-Barr virus infection. Hum Pathol. 1995;26(11):1278–82. 7590705 10.1016/0046-8177(95)90206-6

[17] Marafini I Monteleone G Stolfi C. Association between celiac disease and cancer. Int J Mol Sci. 2020;21(11):4155. 32532079 10.3390/ijms21114155 PMC7312081

[18] Kenwright S. Coeliac disease and small bowel carcinoma. Postgrad Med J. 1972;48(565):673–7. 4650781 10.1136/pgmj.48.565.673 PMC2495314

[19] Aparicio T Zaanan A Svrcek M . Small bowel adenocarcinoma: Epidemiology, risk factors, diagnosis and treatment. Dig Liver Dis. 2014;46(2):97–104. 23796552 10.1016/j.dld.2013.04.013

[20] Vanoli A Di Sabatino A Furlan D . Small bowel carcinomas in coeliac or Crohn's disease: Clinico-pathological, molecular, and prognostic features. a study from the Small Bowel Cancer Italian Consortium. J Crohns Colitis. 2017;11(8):942–53. 28333239 10.1093/ecco-jcc/jjx031

[21] Song HJ Srivastava A Lee J . Host inflammatory response predicts survival of patients with Epstein-Barr virus-associated gastric carcinoma. Gastroenterology. 2010;139(1):84–92.e2. 20398662 10.1053/j.gastro.2010.04.002

[22] van Beek J zur Hausen A Klein Kranenbarg E . EBV-Positive gastric adenocarcinomas: A distinct clinicopathologic entity with a low frequency of lymph node involvement. J Clin Oncol. 2004;22(4):664–70. 14966089 10.1200/JCO.2004.08.061

[23] Chang MS Lee HS Kim CW . Clinicopathologic characteristics of EBV incorporated gastric cancer in Korea. Patho Res Pract. 2001;197:395–400. 10.1078/0344-0338-00052 11432666

